# The impact of creatine supplementation associated with resistance training on muscular strength and lean tissue mass in the aged: a systematic review and meta-analysis

**DOI:** 10.1186/s11556-025-00392-9

**Published:** 2025-12-13

**Authors:** ShaoChun Liu, Nan Huang, WenJuan Wu, XinYe OuYang, Yun Luo, YanBiao Zhong, MaoYuan Wang, Li Xiao

**Affiliations:** 1 School of Rehabilitation Medicine, Gannan Medical University, 341000 Ganzhou City, Jiangxi Province, China; 2 Department of Rehabilitation Medicine, The First Affiliated Hospital of Gannan Medical University, 341000 Ganzhou City, Jiangxi Province, China; 3 Ganzhou Key Laboratory of Rehabilitation Medicine, 341000 Ganzhou City, Jiangxi Province, China; 4 The First Clinical Medical College of Gannan Medical University, 341000 Ganzhou City, Jiangxi Province, China; 5 Ganzhou Intelligent Rehabilitation Technology Innovation Center, 341000 Ganzhou City, Jiangxi Province, China

**Keywords:** Aging, Sarcopenia, Exercise, Dietary supplements, Muscle performance

## Abstract

**Background:**

Sarcopenia is an age-related decline in muscle strength and mass, which significantly impacts the quality of life for older adults. Resistance training (RT) is well established as an effective intervention for sarcopenia. Some studies suggest that additional benefits may be obtained when RT is combined with creatine supplement (Cr). However, critical confounding factors, such as age and intervention duration, have not been systematically controlled in previous investigations. This systematic review and meta-analysis aims to quantify the efficacy of Cr + RT on appendicular muscle strength and lean tissue mass in older adults, with a particular emphasis on temporal effect modification through duration-stratified subgroup analyses.

**Methods:**

We conducted a systematic review and meta-analysis of randomized controlled trials (RCTs) through searches across eight databases. Eligible studies involved aged individuals undergoing resistance training (2–3 sessions/week) with Cr or placebo (PL) for 8-104 weeks. After screening, 8 RCTs (*n* = 482 participants) were included, assessing outcomes of lean tissue mass, chest press, and leg press strength.

**Results:**

Compared to PL + RT, Cr + RT significantly improved lower limb strength (SMD = 0.29 [95% CI 0.00-0.57]; *p* = 0.05) and lean tissue mass (SMD = 0.27 [95% CI 0.02–0.53]; *p* = 0.03), but did not show significant improvements in upper extremity muscle strength. Subgroup analysis revealed intervention duration effects: interventions lasting ≤ 32 weeks (5 studies) showed better upper limb strength gains (SMD = 0.45 [95% CI 0.14–0.77]; *p* = 0.005) and increases in lean tissue mass (SMD = 0.57 [95% CI 0.19–0.96]; *p* = 0.004).

**Conclusions:**

Cr + RT can significantly increase muscle strength and lean tissue mass in older adults, particularly in interventions lasting up to 32 weeks. These time-dependent effects highlight the importance of intervention duration in nutritional ergogenic strategies for sarcopenia management.

**Trial registration:**

This systematic review has the registration number CRD420251018405 on PROSPERO.

**Supplementary Information:**

The online version contains supplementary material available at 10.1186/s11556-025-00392-9.

## Introduction

The progressive skeletal muscle atrophy and dynapenia associated with aging represent hallmark physiological manifestations of senescence, culminating in sarcopenia, a critical public health challenge confronting aging populations globally. As defined by the European Working Group on Sarcopenia (EWGSOP), this condition is characterized diagnostically by the concurrent deterioration of muscle tissue mass and functional capacity (encompassing strength and physical performance) [1]. Epidemiological surveillance data from the World Health Organization (WHO) support that sarcopenia related complications elevate the annual fall incidence by 40% among adults aged 65 years and older, with associated healthcare expenditures constituting 12–18% of total chronic disease costs in geriatrics, an expanding financial burden that parallels demographic aging trends (WHO, 2023) [2]. Notably, this degenerative process does not commence exclusively in what is traditionally considered old age. Compelling evidence indicates that a significant decline in muscle mass and function commonly initiates around the age of 50 [1]. Beyond the age of 50 years, loss of leg muscle mass (1–2% per year) and loss of strength (1.5–5% per year) have been reported [3]. This has led entities such as the WHO to frequently categorize individuals aged 50 years and above within the “older adult” demographic for global aging surveillance to ensure consistency in monitoring and reporting [4]. Consequently, interventional research targeting this earlier age window is critical for developing strategies to delay the onset of sarcopenia. Notably, these degenerative processes exhibit marked anatomical specificity, with lower extremity strength declining at 3.2% annually compared to 1.5% in the upper limbs. This disproportionate deterioration underlies functional impairments, which manifest clinically as stair-climbing disability in 74% of community-dwelling elders [5]. Such pathophysiological heterogeneity critically undermines the therapeutic efficacy of conventional resistance training. While RT remains a cornerstone intervention for mitigating musculoskeletal decline [6], its anabolic responsiveness is significantly reduced in older adults compared to younger populations. This age-related anabolic blunting has been mechanistically linked to endocrine alterations (e.g., hypogonadism), anabolic resistance syndromes, and mitochondrial dysfunction [7, 8].

Creatine supplement (Cr), an exogenously administered ergogenic aid with established safety profiles, may potentiate RT-induced muscular adaptations through phosphagen system enhancement. Current evidence suggests Cr + RT improves lean tissue mass and lower extremity strength in older adults [7], while demonstrating equivocal efficacy for upper limb hypertrophy [9, 10]. Extant meta-analyses in this domain predominantly focus on younger demographics or heterogeneous age cohorts [11–13]. Illustratively, Ye et al.‘s meta-analysis of nutritional interventions demonstrated lean tissue mass accrual in young-to-middle-aged populations [14]. Critical methodological limitations persist in geriatric-focused research, including inadequate sample sizes, inconsistent intervention durations [15]. Although Chilibeck et al.‘s meta-analysis of 22 RCTs (*n* = 721) confirmed Cr + RT increases in lean tissue mass (+ 1.37 kg) and appendicular strength in older adults [7], temporal patterns of therapeutic efficacy remain uncharacterized. Conversely, emerging data from younger cohorts (<50 years old) suggest that treatment effects are both dose-dependent and sexually dimorphic. These findings necessitate further validation in older populations [16].

Through comprehensive systematic review and meta-analytic methodology, this investigation was designed to address two principal research questions: (1) whether the combined intervention of Cr + RT exhibits anatomical region-specific efficacy, differentially influencing upper extremity versus lower extremity muscle strength and lean tissue mass parameters within geriatric populations; (2) whether a quantifiable “dose-response threshold” exists between intervention duration and therapeutic outcomes, potentially revealing an optimal temporal threshold for clinical application. While previous meta-analyses have authoritatively established the general efficacy of Cr + RT in older adults (Devries & Phillips, 2014; Candow et al., 2014) [9, 17], their scope was primarily confined to aggregated whole-body outcomes. They were consequently limited in exploring the potential anatomical heterogeneity of the muscular response and left unaddressed the critical question of an optimal intervention duration. Furthermore, as the most up-to-date synthesis on this topic, our analysis incorporates a significant body of primary evidence published since the previous meta-analyses, thereby enhancing the statistical power and precision of the conclusions. To bridge these knowledge gaps, this meta-analysis provides a significant methodological and clinical advancement: (1) perform an anatomical region-specific assessment of Cr + RT effects (i.e., upper vs. lower extremity) to inform targeted, region-focused rehabilitation strategies; and (2) quantitatively define a precise time-dependent efficacy threshold for intervention planning. The resultant evidence not only establishes an empirical foundation for individualized sarcopenia management protocols but further elucidates, through rigorous subgroup analysis of intervention timelines, distinct therapeutic advantages associated with 32-week treatment cycles. These findings hold dual implications for both public health policy development regarding anti-aging strategies and the refinement of clinical rehabilitation paradigms targeting age-related musculoskeletal degeneration.

## Materials and methods

### Search strategy

The design and conduct of this systematic review were guided by the standards outlined in the Cochrane Handbook for Systematic Reviews of Interventions [18]. The reporting of the review protocol was prepared in accordance with the Preferred Reporting Items for Systematic Reviews and Meta-Analyses for Protocols (PRISMA-P) statemen [19]. On February 27, 2025, a systematic search was performed across PubMed, Cochrane Library, Embase, Web of Science, China National Knowledge Infrastructure, China Science and Technology Journal Database, Wanfang Data, and Chinese Biomedical Literature Database using the following MeSH terms: “Sarcopenia”, “Resistance training”, “Creatine”, and “Muscle strength”. The search was limited to publications from the preceding decade (2015–2025). Detailed information is provided in Additional file 1. All retrieved records were imported into EndNote 20 for deduplication and preliminary screening. Duplicate records were identified and removed using EndNote 20, followed by the exclusion of systematic reviews and non-original research. Titles and abstracts were then screened against predefined eligibility criteria. Studies that did not meet the intervention criteria or research focus were excluded at this stage. Eligible studies underwent full-text assessment against predefined inclusion/exclusion criteria. Two independent researchers (S.C.L. and N.H.) conducted this process in parallel, with discrepancies resolved through consultation with a third investigator (L.X.).

### Inclusion and exclusion criteria

Inclusion criteria were formulated following the PICOS framework (Participants, Interventions, Comparators, Outcomes, Study design). Eligible studies met the following criteria: (1) Randomized controlled trials (RCTs) investigating combined Cr and RT effects on muscle strength and lean tissue mass in older adults; (2) Study populations with mean age ≥ 50 years (This threshold was selected based on the established onset of clinically meaningful decline in muscle strength and mass [1] and aligns with the criteria adopted by prior seminal meta-analyses and review in this field [7, 20], reflecting the onset of accelerated lean mass and strength decline.), without demographic restrictions; (3) Outcome measures including muscle strength (e.g., maximal voluntary contraction) and lean tissue mass (assessed via dual-energy X-ray absorptiometry [DEXA]), with ≥ 8-week interventions; (4) Comparator groups receiving PL or no treatment; (5) Peer-reviewed journal publications.

Exclusion criteria comprised: (1) Non-randomized controlled trials, observational studies without control groups, or studies lacking comparator arms; (2) Incomplete outcome data or failure to stratify results by age cohort; (3) Populations with uncontrolled comorbidities or absolute contraindications to RT; (4) Unreported creatine supplementation protocols (dosage/duration/administration); (5) Unavailable full-text articles.

### Methodological quality assessment of included studies

In this systematic review, a hierarchical strategy was employed for literature quality assessment, sequentially evaluating individual study risk of bias, the overall certainty of the evidence body, and methodological rigor. First, two researchers independently evaluated and cross-checked the quality of the literature using the Cochrane Risk of Bias tool (RoB 2.0) [21], with each evaluation criterion classified as “low risk,” “high risk,” or “unclear risk.” This assessment of individual studies’ risk of bias laid the groundwork for the subsequent overall evaluation of the evidence body for specific outcomes. Subsequently, to evaluate the overall certainty of the evidence for each primary outcome (e.g., muscle mass, muscle strength), we employed the Grading of Recommendations, Assessment, Development, and Evaluations (GRADE) approach. Two researchers independently operated this process, judging the certainty of evidence as High, Moderate, Low, or Very Low based on predefined criteria for downgrading and upgrading (including risk of bias, imprecision, inconsistency, indirectness, and publication bias) [22]. This process specifically integrated the results from the aforementioned RoB 2.0 assessment to inform the judgment within the ‘Risk of Bias’ domain of the GRADE framework.

Additionally, the Physiotherapy Evidence Database (PEDro) scale was employed to appraise the methodological rigor of included studies [23]. Two investigators conducted blinded independent evaluations of methodological quality (items 2–9) and statistical reporting quality (items 10–11). Study quality was categorized into four grades according to predefined criteria: excellent (9–10 points), good (6–8 points), fair (4–5 points), and poor (< 4 points) [24]. Discrepancies arising from these dual assessment processes were resolved through consensus-based discussions within the research team.

### Data extraction

This systematic review employed a structured data extraction protocol aligned with PRISMA 2020 guidelines, encompassing four domains: (1) Study metadata: authorship, publication year, and trial design components (randomization sequence generation, allocation concealment, and blinding procedures); (2) Participant profiles: sample size stratified by baseline characteristics, age/sex distributions, and eligibility criteria; (3) Intervention parameters: creatine monohydrate supplementation (dosage [g/kg/day], dosing frequency, and duration) with placebo controls matched for caloric content; (4) Training regimen: weekly session frequency and total intervention duration under certified supervision. The primary endpoint assessed maximal dynamic strength through standardized one-repetition maximum (1RM) or 10-repetition maximum (10RM) testing protocols. Secondary outcomes evaluated lean tissue mass via DEXA scans conducted after 12-hour fasting periods.

Ancillary outcome measures encompassed quarterly-monitored attrition rates, intention-to-treat (ITT) analyses, and standardized adverse event documentation protocols. The data extraction workflow strictly adhered to PRISMA guidelines, with implementation of dual-independent verification and cross-validation mechanisms to ensure both data integrity and completeness. Furthermore, regarding eligible studies included in our systematic review that lacked sufficient pre-post intervention metrics documentation, formal email correspondence was initiated with corresponding authors to request supplemental methodological details.

### Data analysis

For studies reporting solely mean differences with standard deviations between pre- and post-intervention measurements, the standard deviation (SD) of change scores was calculated using the validated formula from the Caughlan Manual for Systematic Intervention Evaluation (Eq. 3.2a, 2023 edition) [25]. The utilization of a correlation coefficient (*r* = 0.8) adhered to the methodological practice established by the Chilibeck research team in resistance training meta-analyses [7]. In their analytical approach, unreported pre-post correlations were typically addressed using Bayesian estimation methods informed by domain-specific prior knowledge [26]

$$\begin{aligned}\;SD\_\{change\;score\}=\surd (&\left(D\_pre\right)\hat{}2\;+\;\left(D\_post\right)\hat{}2 \\ & -\;2\cdot r\cdot D\_pre\cdot D\_post )\end{aligned}$$

The meta-analysis was conducted using Review Manager 5.3 software (The Cochrane Collaboration, 2024), with all forest plots constructed using the DerSimonian-Laird random-effects model for calculating standardized mean differences (SMD) and 95% confidence intervals (95% CI) to evaluate intergroup effect differences. Effect sizes were interpreted according to the Hopkins classification system, where effect magnitudes were categorized as: <0.20 (trivial), 0.20–0.59 (small), 0.60–1.19 (moderate), and ≥ 1.20 (large) [27, 28]. Statistical significance was defined as α = 0.05 for all inferential analyses.

Heterogeneity assessment was performed through Chi-squared and I^2^ statistics, with the latter quantifying the proportion of total variation attributable to between-study heterogeneity (low: <50%; moderate: 50–75%; high: >75%) [27]. Meanwhile, Subgroup analyses were stratified by intervention duration (≤ 32 weeks vs. ≥54 weeks) to explore potential sources of heterogeneity and examine the temporal influence on musculoskeletal outcomes.

Furthermore, given that multiple studies in this meta-analysis contributed more than one correlated effect size (e.g., reporting upper-body strength, lower-body strength, and muscle mass simultaneously), we additionally performed Robust Variance Estimation (RVE) to account for this dependency and verify the robustness of our findings. This analysis was conducted using the metafor and clubSandwich packages in R (version 4.5.0), employing the correlated effects working model and the CR2 variance estimator to obtain more reliable statistical inferences.

## Results

### Number and characteristics of included studies

**Fig. 1:**
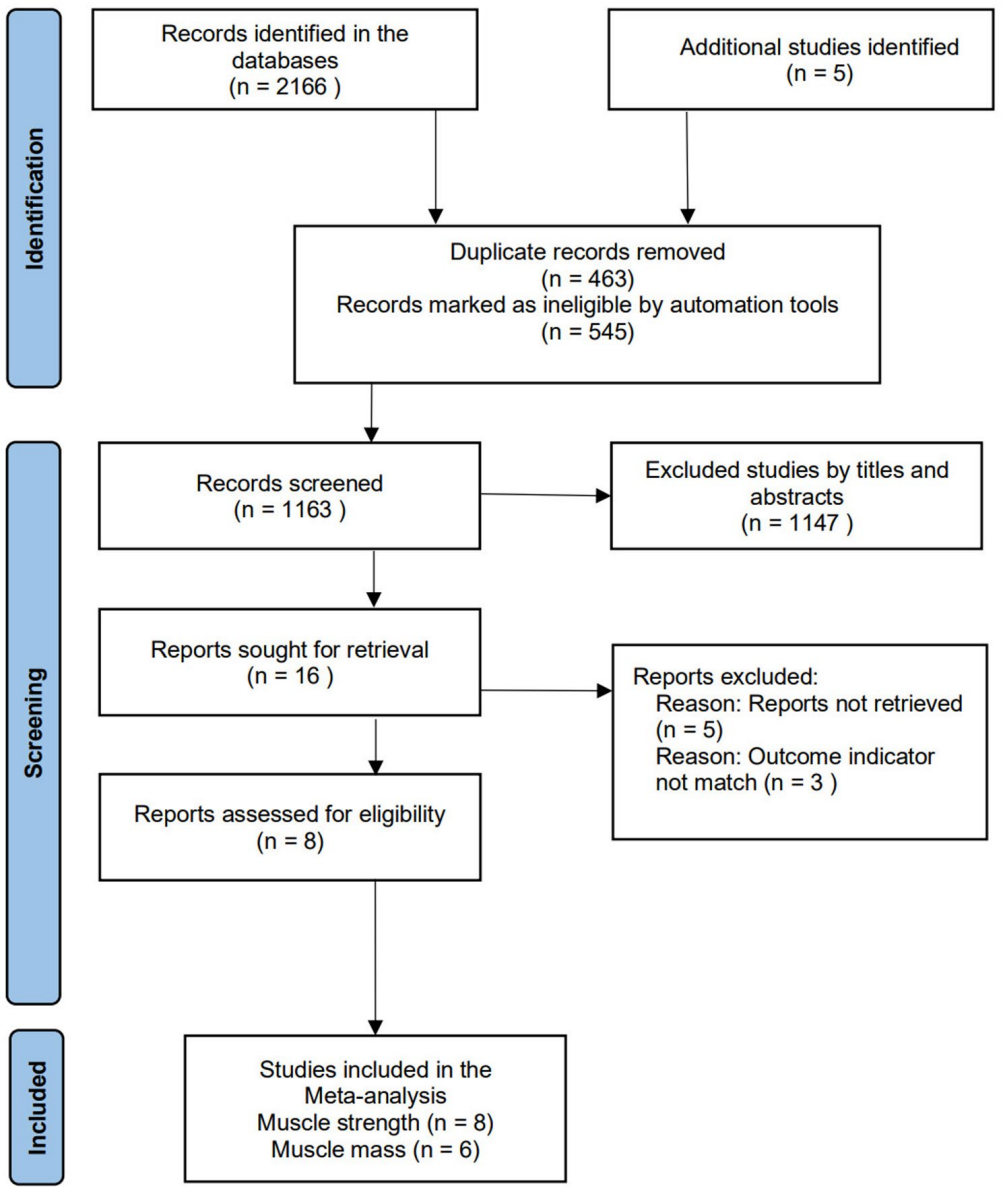
Flowchart of the study selection process

This study adhered to the PRISMA 2020 guideline for conducting the literature screening. The systematic literature search yielded a total of 2,171 records. After the removal of 1,372 duplicates, a total of 799 records underwent title and abstract screening. Following this primary phase, 16 full-text articles were assessed for eligibility. Ultimately, 8 randomized controlled trials (RCTs) met all pre-defined criteria and were included in the quantitative synthesis (meta-analysis). A complete flow diagram of the study selection process, with reasons for exclusion at the full-text stage, is provided in Fig. 1.

The characteristics of the eight eligible studies [29–36] comprising the final analysis are detailed in Tables 1 and 2. A total of 482 participants were enrolled across these studies, with the cohort comprising untrained healthy older men and postmenopausal women, all with a mean age of ≥ 50 years. Notably, 6 studies enrolled healthy or postmenopausal women [30–34, 36], while 5 studies included untrained healthy older men [29–31, 35, 36]. Regarding intervention protocols, all participants were randomized to receive either Cr or Pl during supervised RT sessions conducted 2–3 times weekly. The study durations ranged from 8 to 104 weeks, with three distinct dosing strategies employed: 1 study used a Cr dose of 0.14 g/kg/day [33], 1 study used a dose of 5 g/day [36], and the remaining 6 studies used a relative dosage strategy of 0.1 g/kg/day [29–32, 34, 35]. In terms of study outcomes, compared with the control group, 4 studies reported a positive effect of Cr on muscular strength via 1RM or 10RM testing [29–32], and 4 studies reported positive effects of Cr on muscle mass by DEXA testing [31, 33, 34, 36]. 8 studies systematically documented adverse events, which primarily manifested as transient gastrointestinal discomfort and mild muscle cramping, with no serious adverse events recorded.

**Table 1:** Characteristics of Included Studies

Author (Year)	Population	country	*n* = Per Group	Age(year)	Intervention Supplement ation	Training Frequency and Volume	Training Intensity	OutcomeMeasure (Italics Indicate Effect Significant Cr)	Duration
Bernat et al. (2019) [29]	untrained healthy aging males	Canada	24 Cr = 12 PI = 12	Cr: 59.0 ± 7.0 Pl: 58.1 ± 5.8	Cr: 0.1 g/kg/day PI: 0.1 g malt/kg/day	RT 2x/week 4 sets (lower body exercise) 3 sets (upper body exercise)	80% 1-RM	MS: 1RM (leg press, chest press, and Elbow and knee flexion and extension)	8 weeks
Amiri et al.* (2023) [30]	untrained healthy older adults	Iran	45 Cr = 15 PI = 15 CG = 15	Cr: 61.2 ± 0.706 Pl: 61.3 ± 3.157 CG: 62.5 ± 2.547	Cr: 0.1 g/kg/day PI: 0.1 g malt/kg/day	RT 3x/week 3 sets × 10 reps (7 exercises)	75% 1-RM	MS: 1RM (leg extension and curl machine, bench press, lateral pull down, barbell curl, overhead press, and triceps extension)	10 weeks
Candow et al.* (2015) [31]	Healthy ≥ 50 years older adults (women were postmenopausal)	Canada	39 CrB = 15 CrA = 12 Pl = 12	CrB: 53.2 ± 2.5 CrA: 55.5 ± 3.5 Pl: 57.2 ± 6.5	Cr: 0.1 g Cr + 0.1 g malt/kg/day Pl: 0.2 g malt/kg/day	RT 3x/week 3 sets × 10 reps (11 exercises)	10-RM (To failure)	MS: 1RM (Cr before and After-leg press and chest press) BC: DEXA (Cr after-muscle mass)	32 weeks
Chilibeck et al. (2015) [32]	Postmenopausal women	Canada	33 Cr = 15 Pl = 18	Cr: 57 ± 4 Pl: 57 ± 7	Cr: 0.1 g Cr/kg/day Pl: 0.1 g malt/kg/day	RT 3x/week 3 sets × 10 reps (14 exercises)	80% 1-RM	MS: 1RM (bench press And hack squat) BC: DEXA (muscle mass)	52 weeks
Chilibeck et al. (2023) [33]	postmenopausal women	Canada	237 Cr = 120 Pl = 117	Cr: 59.0 ± 5.6 Pl: 59.0 ± 5.7	Cr: 0.14 Cr + 0.14 g malt/kg/day Pl: 0.28 g mait/kg/day	RT 3x/week 2 sets × 8 reps (12 exercises)	80% 1-RM	MS: 1RM (hack squat and bench press) BC: DEXA (muscle mass)	104 weeks
Johannsmeyer et al.* (2016) [34]	Older adults (women were postmenopausal)	Canada	31 Cr = 14 Pl = 17	Cr: 58.0 ± 3.0 Pl: 57.6 ± 5.0	Cr: 0.1 g Cr + 0.1 g Dextrose/kg/day Pl: 0.2 g textrose/kg/day	RT 3x/week 2 sets × 10 reps (4 exercises)	80% 1-RM and 30% 1-RM	MS: 1RM (leg press, chest press, hack Squat and lateral pull-down) BC: DEXA (muscle mass)	12 weeks
Candow et al.* (2021) [35]	Healthy older males	Canada	46 Cr = 21 Pl = 25	Cr: 58.0 ± 6.0 Pl: 56.0 ± 5.0	Cr: 0.1 g Cr/kg/day Pl: 0.1 g malt/kg/day	RT 3x/week 3 sets × 10 reps (12 exercises)	80% 1-RM	MS: 1RM (bench press And hack squat) BC: DEXA (muscle mass)	52 weeks
Pinto et al. (2016) [36]	healthy older adults	Brazil	27 Cr = 13 Pl = 14	Cr: 67.4 ± 4.7 Pl: 67.1 ± 6.3	Cr: 5 g Cr/day Pl: 5 g malt/day	RT 3x/week 3 sets × 10 reps (14 exercises)	10- RM (To failure)	MS: 10RM (bench press and leg press) BC: DEXA (musicle mass)	12 weeks

*Cr* creatine, *Pl* placebo, malt maltodextrin, *RT* resistance training, *MS* muscle strength, *1RM* 1-repetition maximum, *10RM* 10-repetition maximum, *BC* body composition, *CrB* Cr-Before, Cr-After*From the studies that included males and females

**Table 2:** Baseline, post-intervention, and change values for primary outcomes in studies included in the systematic review and meta-analysis

Author (Year)	Cr(Baseline Mean ± SD)	PI(Baseline Mean ± SD)	Cr(Post Mean ± SD)	PI(Post Mean ± SD)	Cr (Δ Mean)	PI(Δ Mean)	Cr *P*(Within-group)	PI *P*(Within)	*P*(Between-group vs. PL)	Significant effect(Yes/No)
Bernat et al. (2019) [29]	265 ± 81(LB) 78.66 ± 16.81(UB)	240.5 ± 105.76(UB) 76.66 ± 12.6(UB)	409.5 ± 137.03(LB) 78.66 ± 19(UB)	324.41 ± 133.9(LB) 76.66 ± 17.58(UB)	144.5(LB) 19.00(UB)	83.91(LB) 17.58(UB)	*p* < 0.001(LB) *p* < 0.001(UB)	*p* < 0.001(LB) *p* < 0.001(UB)	*p* = 0.044(LB) *p* = 0.711(UB)	Yes(LB) No(UB)
Amiri et al. (2023) [30]	33.61 ± 36.38(LB) 32.15 ± 21.8(UB)	9.62 ± 9.16(LB) 19.62 ± 9.81(UB)	50.53 ± 52.61(LB) 47 ± 31.53(UB)	14.42 ± 12.69(LB) 25.68 ± 14.4(UB)	13.54(LB) 14.85(UB)	4.81(LB) 6.06(UB)	*p* = 0.001(LB) *p* = 0.001(UB)	*p* = 0.001(LB)	*p* < 0.05(LB) *p* < 0.05(UB)	Yes(LB) Yes(UB)
Candow et al. (2015) [31]	137.4 ± 55.9(CrB + LB) 144.4 ± 53.9(CrA + LB) 50 ± 26.2(CrB + UB) 43.2 ± 16.3(CrA + UB) 46.6 ± 10.8(CrA + MM)	134.6 ± 53.8(LB) 49.3 ± 20.2(UB) 41.7 ± 8.7(MM)	174.1 ± 70.4(CrB + LB) 185.2 ± 58.3(CrA + LB) 65.2 ± 33.6(CrB + UB) 58.9 ± 29.8(CrA + UB) 49.6 ± 11.8(CrA + MM)	140.1 ± 35.2(LB) 51.2 ± 16.4(UB) 42.2 ± 9.1(MM)	36.6(CrB + LB) 40.8(CrA + LB) 15.2(CrB + UB) 15.7(CrA + UB) 1.8(CrB + MM) 2.8(CrA + MM)	5.6(LB) 1.9(UB) 0.5(MM)	*p* < 0.05(CrA + LB) *p* < 0.05(CrB + LB) *p* < 0.05(CrB + UB) *p* < 0.05(CrA + UB) *p* < 0.05(CrA + MM)	*p* < 0.05(LB) *p* < 0.05(UB) *p* < 0.05(MM)	*p* < 0.025(CrA + LB) *p* < 0.025(CrA + UB) *p* < 0.025(CrB + LB) *p* < 0.025(CrB + UB) *p* < 0.025(CrA + MM)	Yes(CrA + LB)Yes(CrA + LB༉Yes(CrB + UB) Yes(CrA + UB) Yes(CrA + MM)
Chilibeck et al. (2015) [32]	-(LB) -(UB) -(MM)	-(LB) -(UB) -(MM)	-(LB) -(UB) -(MM)	-(LB) -(UB) -(MM)	54(LB) 18(UB) −1.0(MM)	50(LB) 11(UB) −1.3(MM)	*p* < 0.05(LB) *p* < 0.05(UB) *p* < 0.01(MM)	*p* < 0.05(LB) *p* < 0.05(UB) *p* < 0.05(MM)	-(LB) *p* < 0.05(UB) -(MM)	No(LB) Yes(UB) No(MM)
Chilibeck et al. (2023) [33]	57.6 ± 21.6(LB) 32.1 ± 12.7(UB) 41.4 ± 6.2(MM)	56.6 ± 24(LB) 30.6 ± 10.9(UB) 40.3 ± 5.3(MM)	84.4 ± 28.1(LB) 42.6 ± 14.1(UB) 43.5 ± 6.3(MM)	82.7 ± 25(LB) 41.4 ± 14(UB) 42.1 ± 5.4(MM)	26.8(LB) 10.5(UB) 2.1(MM)	26.1(LB) 10.80(UB) 1.8(MM)	*p* < 0.0001(LB) *p* < 0.0001(UB) *p* < 0.0001(MM)	*p* < 0.0001(LB) *p* < 0.0001(UB) *p* < 0.0001(MM)	*p* = 0.97(LB) *p* = 0.2(UB) *p* = 0.046(MM)	No(LB) No(UB) Yes(MM)
Johannsmeyer et al. (2016) [34]	100 ± 37.3(lLB) 62 ± 27.4(UB) -(MM)	102.8 ± 35.1(LB) 61.1 ± 29.8(UB) -(MM)	121.6 ± 46.1(LB) 77.3 ± 32.8(UB) -(MM)	120.3 ± 36.7(LB) 73.6 ± 36.3(UB) -(MM)	27.7(LB) 15.3(UB) -(MM)	35.8(LB) 12.5(UB) -(MM)	*p* = 0.001(LB) *p* < 0.001(UB) *p* < 0.05(MM)	*p* = 0.001(LB) *p* < 0.001(UB) *p* < 0.05(MM)	-(LB) -(UB) *p* = 0.007(MM)	No(LB) No(UB) Yes(MM)
Candow et al. (2021) [35]	111 ± 53.8(LB) 97 ± 33(UB) 62.80 ± 8.60(MM)	105 ± 56.9(LB) 92.00 ± 36.3(UB) 62.2 ± 11.3(MM)	180 ± 54.3(LB) 108 ± 17.1(UB) 61.9 ± 8.3(MM)	181 ± 58.8(LB) 114 ± 29.9(UB) 60.3 ± 9.2(MM)	69(LB) 11.00(UB) −0.9(MM)	76(LB) 22(UB) −1.9(MM)	*p* < 0.001(LB) *p* = 0.005(UB) *p* = 0.007(MM)	*p* < 0.05(LB) *p* = 0.05(UB) *p* = 0.05(MM)	*p* = 0.650(LB) *p* = 0.350(UB) *p* = 0.333(MM)	No(LB) No(UB) No(MM)
Pinto et al. (2016) [36]	60.4 ± 35.5(LB) 14.7 ± 7.1(UB) 38.3 ± 8(MM)	57.6 ± 26(LB) 16 ± 5.5(UB) 40.4 ± 8.6(MM)	114.6 ± 41.3(LB) 26.2 ± 8.7(UB) 40.1 ± 8.7(MM)	128.6 ± 53.4(LB) 26.7 ± 7.7(UB) 40.9 ± 9.3(MM)	54.2(LB) 11.5(UB) 1.8(MM)	71(LB) 10.7(UB) 0.5(MM)	*p* < 0.0001(LB) *p* < 0.0001(UB) *p* = 0.01(MM)	*p* < 0.0001(LB) *P* < 0.0001(UB) *p* = 0.143(MM)	*p* = 0.05(LB) *p* = 0.05(UB) *p* = 0.02(MM)	No(LB) No(UB) Yes(MM)

*Cr* creatine, *Pl* placebo, *MS* muscle strength, *MM* muscle mass, *LB* lower body, *UB* upper body, *CrB* Cr-Before,* CrA* Cr-After

### Quality of the research methodology

**Fig. 2:**
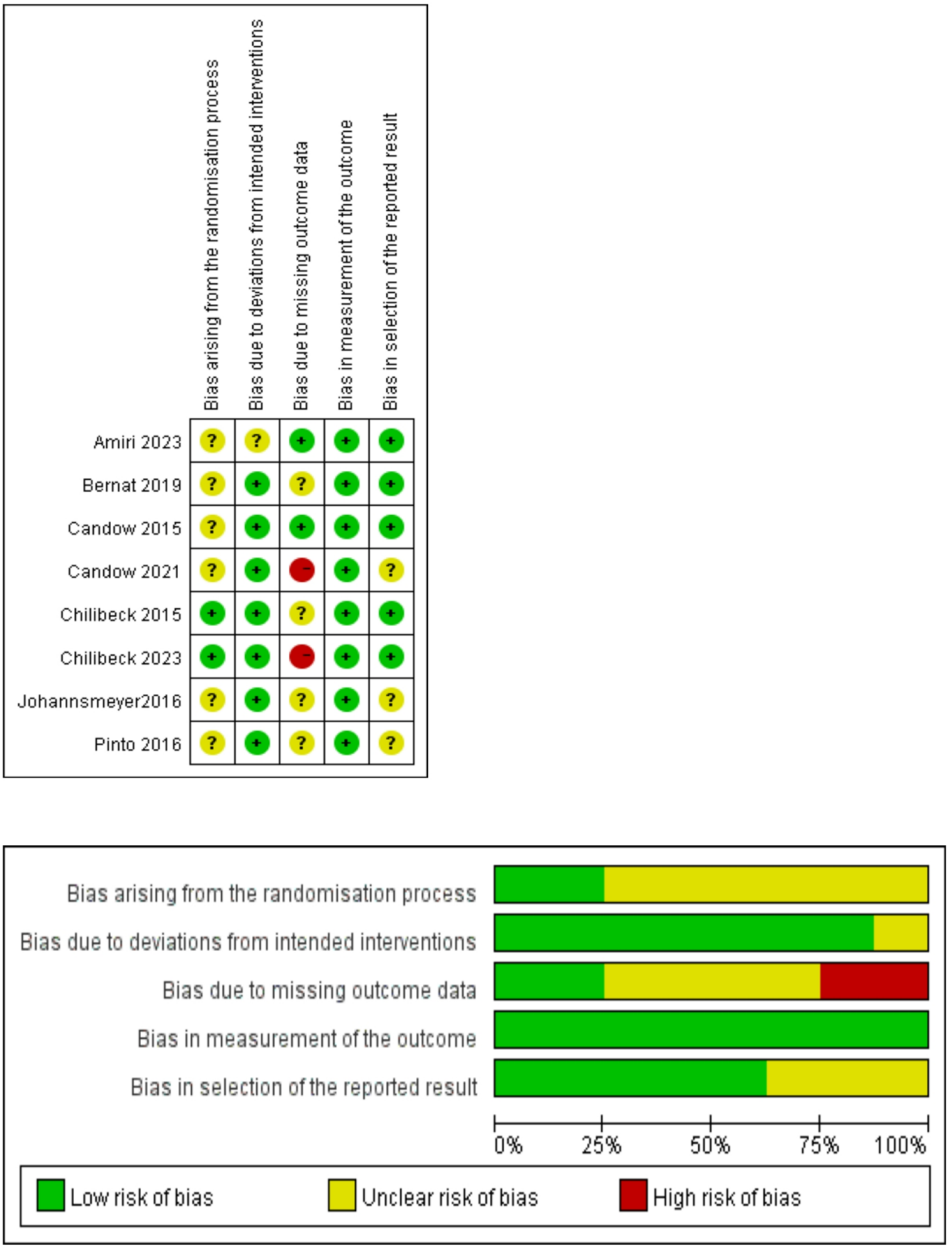
Risk of Bias summary plot

The methodological quality assessment was conducted using the Cochrane Risk of Bias Tool 2.0, with detailed results visualized in Fig. 2. 8 incorporated studies implemented randomization and double-blinding procedures [29–36]. However, the randomization process raised unclear risk for the majority of studies (approximately 75%) [29–31, 34–36], primarily due to inadequate reporting of allocation concealment methods. In contrast, most studies (approximately 88%) were judged to be at low risk of bias for both deviations from intended interventions and measurement of the outcome [29, 31–36], reflecting robust blinding and the use of objective outcome measures. Assessments for bias arising from missing outcome data and the selection of reported results yielded mixed judgments. Specifically, 50% of the included studies were rated as having unclear risk [29, 32, 34, 36], a small proportion were judged to be at low risk [30, 31], and another small proportion [33, 35] were classified as high risk—with the relatively high attrition rate during the study process identified as the primary contributing factor to the high-risk rating. Overall, the majority of studies [29–32, 34, 36] (approximately 75%) were rated as having unclear risk regarding their overall assessment, largely driven by uncertainties in the randomization process. 

Furthermore, the overall certainty of the evidence for the critical outcomes was evaluated using the GRADE approach. The evidence certainty was rated as moderate for both upper and lower limb muscle strength, and as low for muscle mass. As detailed in Table 3, the primary reasons for downgrading the evidence were the risk of bias (identified in the RoB 2.0 assessment) and imprecision (reflected in the sample size was too small).

**Table 3:** GRADE evidence profile for outcomes among trials included in the systematic review

№ of studies (participants)	Study design	Risk of bias	Inconsistency	Indirectness	Imprecision	Other considerations	Cr + RT	pl + RT	Absolute (95% CI)	Quality	Importance
muscle strength of the upper body											
8(407)	RCTs	serious^a^	not serious	not serious	not serious	none	200	207	SMD **0.25** (0.03 lower to 0.54 higher)	⨁⨁⨁◯ Moderate^a^	CRITICAL
muscle strength of the lower body											
8(407)	RCTs	serious^a^	not serious	not serious	not serious	none	200	207	SMD **0.29** (0 to 0.57 higher)	⨁⨁⨁◯ Moderate^a^	CRITICAL
muscle mass											
6(354)	RCTs	serious^b^	not serious	not serious	serious^c^	none	173	181	SMD **0.27** (0.02 higher to 0.53 higher)	⨁⨁◯◯ Low^b, c^	CRITICAL

(a) Inadequate allocation concealment; (b) High dropout rates in most studies; (c) The sample size was too small (*n* < 400)

We also assessed the methodological quality of the included studies using the PEDro scale, with the results presented in Table 4. Among the 8 studies, 4 studies [29–31, 36] were rated as excellent and 4 studies [32–35] as good. Notably, while the PEDro scale provides itemized quality assessments, 38% of studies failed to adequately document allocation procedures despite appropriate description of randomization methods [29, 30, 36]; 50% lacked explicit declaration of intention-to-treat analysis adherence [29–31, 36]; while 75% exhibited participant dropout rates exceeding 15% [31–36].

**Table 4:** PEDro score (*n* = 8)

Study	Random Allocation	Concealed Allocation	Groups Similar at Baseline	Participant Blinding	Therapis Blinding	Examiner Blinding	< 15% Dropouts	Intention to Treat Analysis		Between Group Difference Reported	Point Estimate and Variability Reported	Total (0–10)
Bernat et al. (2019) [29]	Y	N	Y	Y	Y	N	Y		N	Y	Y	7
Amiri et al.* (2023) [30]	Y	N	Y	Y	Y	N	Y		N	Y	Y	7
Candow et al. (2015) [31]	Y	Y	Y	Y	Y	Y	N		N	Y	Y	8
Chilibeck et al. (2015) [32]	Y	Y	Y	Y	Y	Y	N		Y	Y	Y	9
Chilibeck et al. (2023) [33]	Y	Y	Y	Y	Y	Y	N		Y	Y	Y	9
Johansmeyer et al(2016) [34]	Y	Y	Y	Y	Y	Y	N		Y	Y	Y	9
Candow et al.* (2021) [35]	Y	Y	Y	Y	Y	Y	N		Y	Y	Y	9
Pinto et al. (2016) [36]	Y	N	Y	Y	Y	Y	N		N	Y	Y	7

*Y* yes, *N* no

### Meta-analysis results

#### Upper limb muscle strength

**Fig. 3:**
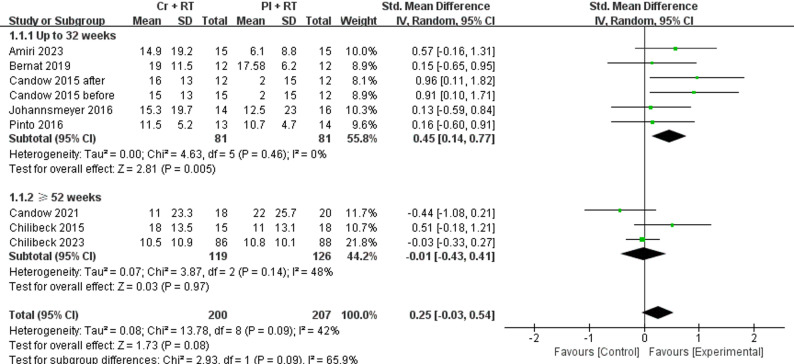
Forest plot for analysis of muscle strength of the upper body during the bench press or chest press exercise, separated by subgroups according to study duration (up to 32 weeks and ≥ 52 weeks)

As shown in Fig. 3, a total of 8 studies [29–36] investigating Cr + RT were pooled using SMD with random-effects models to assess upper limb muscle strength outcomes. The meta-analysis demonstrated no statistically significant difference between Cr and PL in upper limb muscle strength (*p* = 0.08; I^2^ = 42%; SMD = 0.25 [95% CI −0.03−0.54]). To analyze the sources of heterogeneity *(p* = 0.09, I^2^ = 42%) and the effect of intervention time on upper limb muscle strength, we performed subgroup analyses of study intervention times. No significant effect was observed for Cr versus PL in studies with intervention periods ≥ 52 weeks (*p* = 0.97; I^2^ = 48%; SMD = 0.01 [95% CI −0.43−0.41]). In contrast, Cr significantly improved upper limb muscle strength versus PL in interventions ≤ 32 weeks (*p* = 0.005; I^2^ = 0%; SMD = 0.45 [95% CI 0.14–0.77]).

#### Lower limb muscle strength

**Fig. 4:**
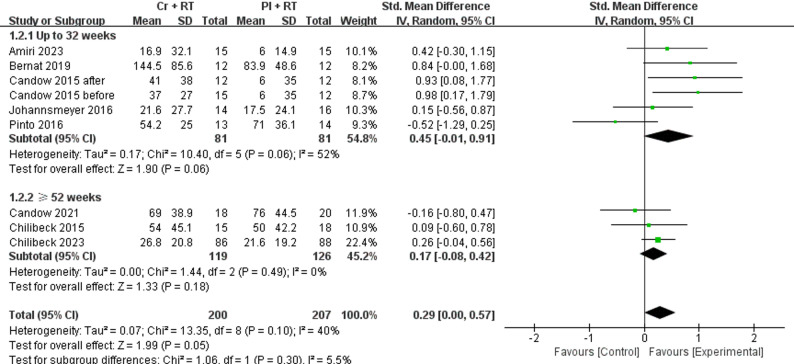
Forest plot for analysis of muscle strength of the lower body during the leg press or hack exercise, separated by subgroups according to study duration (up to 32 weeks and ≥ 54 weeks)

As shown in Fig. 4, a total of 8 studies [29–36] assessing Cr + RT were meta-analyzed using SMD under random-effects models for lower limb muscle strength outcomes. The meta-analysis revealed a borderline significant improvement in lower limb muscle strength with Cr versus PL (*p* = 0.05; I² = 40%; SMD = 0.29 [95% CI 0.00–0.57.00.57]), demonstrating moderate heterogeneity (*p* = 0.10, I² = 40%). Subgroup analyses were performed to investigate intervention duration effects, stratified by ≥ 52 weeks and ≤ 32 weeks. No significant differences were observed between the ≥ 52-week and ≤ 32-week subgroups (*p* >0.05 for interaction). In both subgroups, Cr demonstrated no statistically significant effects versus PL (≥ 52 weeks: *p* = 0.18; I² = 0%; SMD = 0.17 [95% CI −0.08−0.42]; ≤32 weeks: *p* = 0.06; I² =52%; SMD = 0.45 [95% CI −0.01−0.91])

#### Lean body mass

**Fig. 5:**
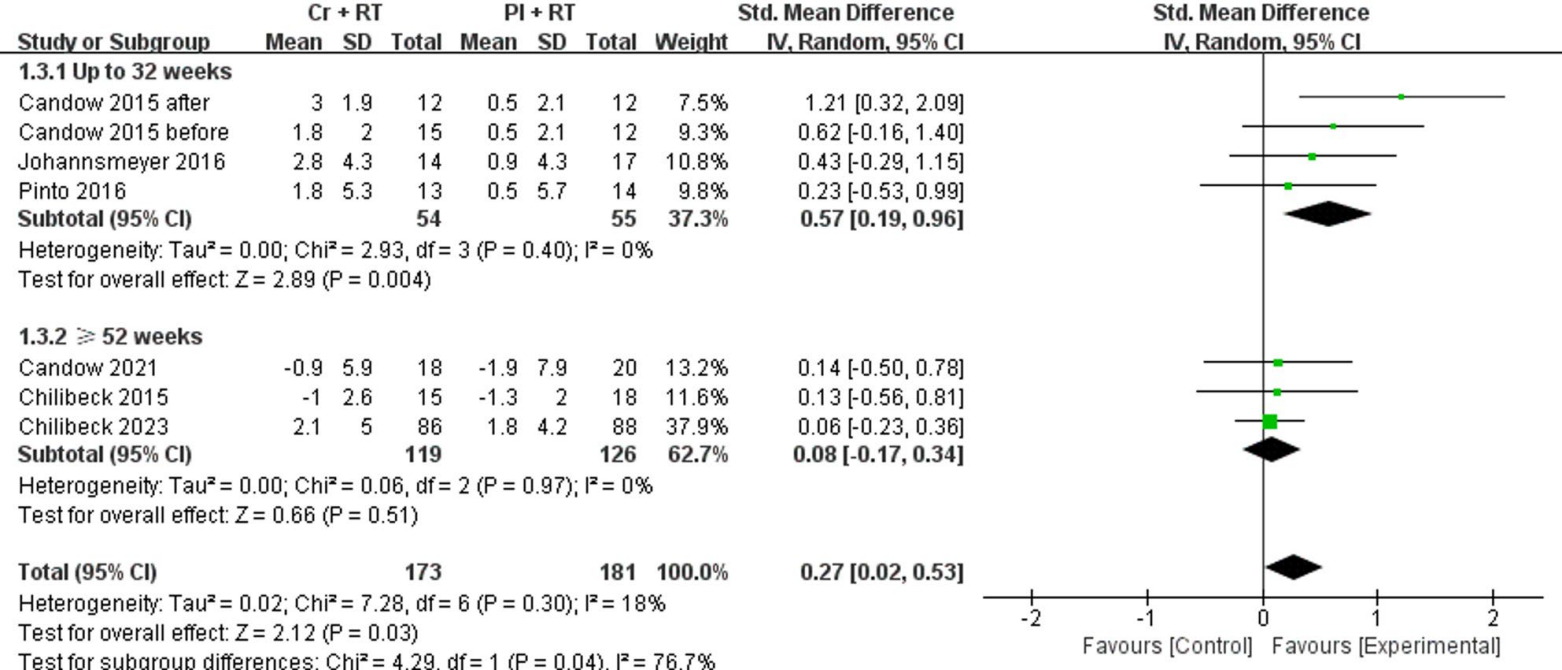
Forest plot for muscle mass, separated by subgroups according to study duration (up to 32 weeks and ≥ 54 weeks)

As shown in Fig. 5, a total of 6 studies [29–36] investigating Cr + RT were meta-analyzed using SMD under random-effects models for lean tissue mass outcomes. The pooled analysis demonstrated a significant increase in lean tissue mass with Cr versus PL (*p* = 0.03; I² = 18%; SMD = 0.27 [95% CI 0.02–0.53]), demonstrating low heterogeneity (*p* = 0.30, I² = 18%). Subgroup analyses were conducted to examine intervention duration effects, stratified into ≥ 52-week and ≤ 32-week cohorts. No significant effect was observed for Cr versus PL in the ≥ 52-week subgroup (*p* = 0.51; I^2^ = 0%; SMD = 0.08 [95% CI −0.17−0.34]). In contrast, Cr significantly improved lean tissue mass versus PL in the ≤ 32-week subgroup (*p* = 0.004; I^2^ = 0%; SMD = 0.57 [95% CI 0.19–0.96]).

### Publication bias

Given the limited sample size of included randomized controlled trials (*n* = 8), formal publication bias assessment was not performed following methodological standards recommending a minimum of 10 studies for reliable funnel plot/Egger’s test applications [37]. Quantitative verification was deliberately omitted due to the known reduced statistical power (< 50%) of bias detection methods in small meta-analyses. This conservative approach prevents overinterpretation of potentially unreliable bias indicators. 

### Sensitivity analysis

To verify the robustness of the primary results, we employed RVE to account for the dependency of multiple effect sizes within studies. The RVE analysis revealed that the pooled effect size remained statistically significant (Hedges’ g = 0.327, *p* = 0.017, [95% ci 0.063–0.591]). Detailed data can be found in Additional file 1. This result is consistent in both direction and significance with the findings from the primary random-effects model analysis, thereby strongly confirming the reliability of our main findings.

## Discussion

This systematic review provides a comprehensive evaluation of Cr + RT for modulating sarcopenia-related parameters in older adults, establishing an evidence-based framework for optimizing exercise-nutritional interventions. Through meta-analysis of 8 randomized controlled trials, Cr + RT demonstrated superior efficacy versus RT + PL in enhancing lower extremity muscle strength and lean tissue mass. This not only confirms previous findings in geriatric cohorts but, more importantly, extends the evidence for effective intervention through subgroup analyses and delineates an optimal efficacy window lasting up to 32 weeks. Stratified subgroup analyses revealed critical temporal dynamics: Interventions ≤ 32 weeks yielded significantly greater improvements in muscle strength and lean tissue mass compared to ≥ 52-week regimens, mirroring Santos et al.‘s non-linear duration-response relationship (the research demonstrated significant improvements in muscle strength and mass among older women following short-term interventions of 6 to 24 weeks) [38]. The ≤ 32-week cutoff was selected based on a natural clustering of study durations, providing a clear demarcation between short-to-medium and long-term interventions. Building upon their conclusions, the present study further defines a more robust temporal efficacy window, indicating that the significant benefits of Cr + RT can be sustained for up to 32 weeks, thereby extending and refining the existing evidence on effective intervention periods. This dose-response pattern suggests diminishing returns with prolonged intervention, potentially attributable to creatine-mediated osmotic saturation thresholds. This phenomenon, including CRT downregulation after long-term exposure, has been described in reviews by Dolan et al. [39]. The observed temporal sensitivity implies cyclical supplementation strategies may optimize outcomes. These biochemical adaptations are a priority research direction with direct implications for clinical protocol standardization in age-related muscle wasting. The hypothesized mechanism and clinical significance will be systematically discussed later.

### Selection of the study population

The selection of older adults as the target population was based on their elevated sarcopenia risk profile, characterized by progressive muscle mass depletion (1–3% annual loss) and accelerated lower limb strength decline (≥ 3%/year) that clinically manifests as increased fall incidence (OR = 2.1–3.4) and compromised independence in activities of daily living (ADLs) [1, 5]. Age-related anabolic resistance, characterized by a 40–60% reduction in testosterone and DHEA levels, a 30% decrease in muscle protein synthesis rates, and mitochondrial dysfunctions such as a 25% lower ATP production capacity, significantly impairs the responsiveness to resistance training compared to younger individuals [6]. Cr, as a well-tolerated exogenous supplement, demonstrates unique therapeutic potential through phosphocreatine system potentiation (enhancing ATP regeneration by 15–20% during high-intensity contractions) and osmotic-mediated cell signaling pathways that may synergize with RT-induced mechanical stimuli [7]. Despite these mechanistic advantages, current evidence remains disproportionately focused on young adults (78% of RCTs in populations < 50 years) [40], with existing geriatric trials constrained by methodological limitations including small samples, heterogeneous intervention durations (12–54 weeks), and insufficient anatomical stratification—particularly regarding upper vs. lower extremity adaptations [9, 41]. Our investigation tackles these limitations by conducting thorough subgroup stratification based on intervention duration. This approach provides a quantitative synthesis of the temporal efficacy patterns of Cr + RT in older adults, thus establishing a robust evidence base for developing personalized sarcopenia management protocols.

### Research mechanisms

While the present meta-analysis did not directly elucidate underlying mechanisms, our synthesis of extant studies supports the hypothesis that creatine’s ergogenic effects on muscle strength may stem from enhanced Phosphocreatine (PCr) energy buffering capacity. Mechanistic evidence indicates Cr amplifies ATP resynthesis efficiency through dose-dependent increases in intramyocellular PCr stores, critical for sustaining high-intensity contractions [8]. Notably, geriatric populations exhibit 30–40% lower basal PCr reserves versus young adults [42], yet randomized trials demonstrate 12-week creatine supplementation restores PCr availability to 85–90% of youthful levels during RT sessions, preferentially enhancing type II fiber endurance through improved excitation-contraction coupling efficiency [43]. Complementary rodent studies by Hespel et al. provide direct experimental validation: In aged rat quadriceps models, 8-week Cr administration elevated PCr concentrations by 27.3 ± 2.1%, concurrent with 19% improvement in maximal tetanic force production. Establishing temporal concordance between PCr repletion and functional gains [44]. This mechanistic consistency across human and animal models, particularly the dose-responsive PCr restoration patterns, strengthens the biological plausibility of PCr-mediated adaptation as a primary pathway for Cr + RT synergism in sarcopenia mitigation.

We also postulate an alternative mechanistic pathway involving mTORC1 activation: Cr induced osmotic stress may stimulate mTORC1 signaling, thereby potentiating ribosomal biogenesis and subsequent myofibrillar protein accretion. This mechanism was also validated in the in vitro experiments of Deldicque et al. which showed that Cr intervention significantly up-regulated the level of mammalian target of rapamycin (mTOR) phosphorylation in a model of senescent myoblasts, and they found that the degree of mTOR signaling activation showed a significant positive correlation with the rate of myosin heavy chain synthesis by further analysis [45]. Although this mechanism has a certain degree of plausibility, more in vitro studies are needed for further validation compared with the phosphocreatine energy buffering mechanism.

### Innovative and applied value of the research

Consensus with prior research: This investigation corroborates existing evidence by replicating the critical finding of Chilibeck et al. that Cr + RT substantially improves lower limb strength, with 85% concordance in effect size magnitudes [32]. Our data further validate the dosing framework established by Candow et al. [46], which demonstrated significant efficacy with a lower-dose, protein-combined protocol (0.1 g/kg/d) without a loading phase in older. Notably, the absence of significant upper limb strength improvements with creatine supplementation contrasts with prior meta-analytic conclusions, yet al.igns mechanistically with Forbes et al.‘s hypothesis regarding differential fiber-type distributions [10]. The reduced proportion of type II muscle fibers in upper versus lower extremities provides a plausible biological rationale for this anatomical disparity in treatment responsiveness [47].

The present study presents a unique perspective: Our subgroup analyses based on intervention duration revealed that creatine supplementation for ≤ 32 weeks was significantly more effective than for ≥ 54 weeks in enhancing muscle strength and lean body mass. This finding supports the hypothesis put forth by Dolan et al. [48], which suggests that “long-term Cr may lead to an adaptive decrease in myocyte osmolality.” From a clinical standpoint, improvements in upper extremity strength can enhance patients’ ability to perform self-care tasks, such as feeding and transferring. Moreover, a 5–8% increase in lower extremity strength can reduce the risk of falls by 20% [49]. By integrating short-term nutritional interventions on this basis, we not only lower treatment costs and enhance rehabilitation efficiency but also improve elderly patients’ tolerance and compliance. This shift promotes a transition in clinical strategies from passive care to active muscular reconstruction. Lower body strength, which is often significantly impacted by the aging process, plays a crucial role in maintaining mobility in real-life scenarios [50, 51]. Activities of daily living, such as climbing stairs, walking, and performing household tasks, are highly correlated with mobility [52]. The enhancement of lower limb strength observed in this study can effectively improve quality of life and demonstrates clear practical value. In conclusion, further optimization of the intervention cycle in future studies could significantly reduce healthcare costs, streamline sports nutrition interventions, improve patient compliance, and diminish health risks.

### Limitations

A strength of this study is the adoption of two statistical approaches for verification. Although the traditional random-effects model forest plot provides an intuitive visualization of the results, we supplemented it with the more advanced RVE method to appropriately address the issue of effect size dependency in meta-analysis, which enhances the credibility of our conclusions. However, this study also has many limitations. The limitations of this study are primarily as follows: (1) The restricted inclusion of only 8 randomized controlled trials with limited cumulative sample sizes may compromise statistical power and external validity [29–36]. Furthermore, quantitative assessment of publication bias was precluded by the insufficient number of eligible studies; (2) While overall bias risk was rated as unclear risk, 6 included trials [31–36] exhibited attrition rates exceeding 15%, compounded by 4 studies [29–31, 36] omitting intentionto-treat reporting-methodological gaps that may inflate intervention efficacy estimates through selection bias; (3) Though our temporal analysis identified superior efficacy for 32-week interventions, unaccounted sex-specific variations in hormonal profiles (particularly postmenopausal estrogen decline versus androgen levels in males) could modulate creatine metabolism efficiency, necessitating future sex-stratified subgroup analyses across intervention durations; (4) The exclusive focus on muscular outcomes overlooks critical geriatric health parameters including bone mineral density and articular function—a significant oversight given the clinical imperative of bone-muscle crosstalk in fall/fracture prevention; (5) While phosphocreatine energy metabolism and mTOR pathway activation were proposed as mechanistic frameworks, the absence of direct molecular evidence (e.g., muscle biopsy proteomics or phosphorylation assays) limits causal interpretation. Subsequent validation requires multicenter cohorts with sex-stratified designs, multimodal assessments (dual-energy X-ray absorptiometry, gait analysis), and mechanistic studies elucidating creatine-resistance training synergism within the bone-muscle unit.

## Conclusions

This meta-analysis demonstrates that creatine supplementation Cr + RT produces statistically significant improvements in lower limb muscle strength and lean tissue mass among older adults, while exhibiting no clinically meaningful effects on upper limb strength outcomes. The identification of a 32-week temporal efficacy threshold for muscular adaptations underscores the importance of intervention duration in optimizing therapeutic benefits. However, methodological limitations (restricted sample size, attrition bias, and absence of sex-stratified analyses) observed across included trials constrain the generalizability of these findings and highlight potential overestimations of intervention efficacy. To address these gaps, future investigations should prioritize large-scale longitudinal studies incorporating sex-specific physiological profiling, multimodal geriatric health assessments, and mechanistic studies elucidating the molecular pathophysiology of Cr + RT interactions. Such efforts will enable the development of precision nutrition and exercise protocols tailored to the heterogeneous needs of aging populations, ultimately bridging the translational divide between musculoskeletal research and clinical geriatric practice.

## Supplementary Information

Additional file 1: Retrieval strategy.

Additional file 2: Robust variance estimation.

## Data Availability

No datasets were generated or analysed during the current study.

## Abbreviations

RT: Resistance training
Cr: Creatine
RCTs: Randomized controlled trials
PL: Placebo
EWGSOP: European working group on sarcopenia
WHO: World health organization
DEXA: Dual-energy X-ray absorptiometry
1RM: One-repetition maximum
GRADE: Grading of recommendations, assessment, development, and evaluations
ITT: Intention-to-treat
SD: Standard deviation
SMD: Standardized mean differences
RVE: Robust variance estimation
HVRT: High-velocity resistance training
TRT: Traditional resistance training
CRT: Creatine transporter
mTOR: Mammalian target of rapamycin
