# Supplementary material for: The impact of creatine supplementation associated with resistance training on muscular strength and lean tissue mass in the aged: a systematic review and meta-analysis
Source: Eur Rev Aging Phys Act. 2025 Dec 13;22:26. doi: 10.1186/s11556-025-00392-9 (PMC12752335; doi:10.1186/s11556-025-00392-9)
Supplement: Supplementary file 1 — Additional file 1: Retrieval strategy. [file 11556_2025_392_MOESM1_ESM.docx]

Appendix 1. Retrieval strategy

pubmed：

| **Search** | **Query** | **Results** |
| --- | --- | --- |
| #13 | Search: ((("Sarcopenia"[Mesh]) OR ((((((strength[Title/Abstract]) OR (lean mass[Title/Abstract])) OR (muscle[Title/Abstract])) OR (muscle mass[Title/Abstract])))) AND ((("Creatine"[Mesh]) OR ("Dietary Supplements"[Mesh])) OR (((creatine supplementation[Title/Abstract]) OR (creatine monohydrate[Title/Abstract])) OR (creatine loading[Title/Abstract]))) AND (("Resistance Training"[Mesh]) OR (((((((((((((((((((((((Training, Resistance[Title/Abstract]) OR (Strength Training[Title/Abstract])) OR (Training, Strength[Title/Abstract])) OR (Weight-Lifting Strengthening Program[Title/Abstract])) OR (Strengthening Programs, Weight-Lifting[Title/Abstract])) OR (Strengthening Program, Weight-Lifting[Title/Abstract])) OR (Weight Lifting Strengthening Program[Title/Abstract])) OR (Weight-Lifting Strengthening Programs[Title/Abstract])) OR (Weight-Lifting Exercise Program[Title/Abstract])) OR (Exercise Programs, Weight-Lifting[Title/Abstract])) OR (Exercise Program, Weight-Lifting[Title/Abstract])) OR (Weight Lifting Exercise Program[Title/Abstract])) OR (Weight-Lifting Exercise Programs[Title/Abstract])) OR (Weight-Bearing Strengthening Program[Title/Abstract])) OR (Strengthening Programs, Weight-Bearing[Title/Abstract])) OR (Strengthening Program, Weight-Bearing[Title/Abstract])) OR (Weight Bearing Strengthening Program[Title/Abstract])) OR (Weight-Bearing Strengthening Programs[Title/Abstract])) OR (Weight-Bearing Exercise Program[Title/Abstract])) OR (Exercise Programs, Weight-Bearing[Title/Abstract])) OR (Exercise Program, Weight-Bearing[Title/Abstract])) OR (Weight Bearing Exercise Program[Title/Abstract])) OR (Weight-Bearing Exercise Programs[Title/Abstract]))) Sort by: Most Recent | 744 |

EMBASE：

#10 #3 AND #6 AND #9 365

#9 #7 OR #8 36,651

#8 'training, resistance' OR 'strength training' OR 'training, strength' OR 'weight-lifting strengthening program' OR 'strengthening programs, weight-lifting' OR 'strengthening program, weight-lifting' OR 'weight lifting strengthening program' OR 'weight-lifting strengthening programs' OR 'weight-lifting exercise program' OR 'exercise programs, weight-lifting' OR 'exercise program, weight-lifting' OR 'weight lifting exercise program' OR 'weight-lifting exercise programs' OR 'weight-bearing strengthening program' OR 'strengthening programs, weight-bearing' OR 'strengthening program, weight-bearing' OR 'weight bearing strengthening program' OR 'weight-bearing strengthening programs' OR 'weight-bearing exercise program' OR 'exercise programs, weight-bearing' OR 'exercise program, weight-bearing' OR 'weight bearing exercise program' OR 'weight-bearing exercise programs' 10,532

#7 'resistance training'/exp 32,489

#6 #4 OR #5 38,444

#5 'dietary supplements' OR 'creatine supplementation' OR 'creatine monohydrate' OR 'creatine loading' 19,002

#4 'creatine'/exp 21,112

#3 #1 OR #2 2,457,502

#2 'strength' OR 'lean mass' OR 'muscle' OR 'muscle mass' 2,452,053

#1 'sarcopenia'/exp 26,881

Cochrane：

#1 MeSH descriptor: [Sarcopenia] explode all trees 977

#2 (Sarcopenias):ti,ab,kw OR (strength):ti,ab,kw OR (lean mass):ti,ab,kw OR (muscle):ti,ab,kw OR (muscle mass):ti,ab,kw (Word variations have been searched) 144165

#3 MeSH descriptor: [Creatine] explode all trees 941

#4 (creatine loading):ti,ab,kw OR (creatine supplementation):ti,ab,kw OR (creatine monohydrate):ti,ab,kw (Word variations have been searched) 2044

#5 MeSH descriptor: [Resistance Training] explode all trees 5781

#6 (Training, Resistance):ti,ab,kw OR (Strength Training):ti,ab,kw OR (Training, Strength):ti,ab,kw OR (Weight-Bearing Exercise Program):ti,ab,kw OR (Weight-Bearing Strengthening Program):ti,ab,kw (Word variations have been searched) 35242

#7 (Weight-Bearing Exercise Programs):ti,ab,kw OR (Exercise Program, Weight-Bearing):ti,ab,kw OR (Strengthening Program, Weight-Bearing):ti,ab,kw OR (Strengthening Programs, Weight-Bearing):ti,ab,kw OR (Weight Bearing Strengthening Program):ti,ab,kw (Word variations have been searched) 623

#8 (Exercise Programs, Weight-Bearing):ti,ab,kw OR (Weight-Bearing Strengthening Programs):ti,ab,kw OR (Weight-Lifting Exercise Programs):ti,ab,kw OR (Weight Lifting Exercise Program):ti,ab,kw OR (Exercise Program, Weight-Lifting):ti,ab,kw (Word variations have been searched) 1183

#9 (Weight-Lifting Exercise Program):ti,ab,kw OR (Weight Lifting Strengthening Program):ti,ab,kw OR (Exercise Programs, Weight-Lifting):ti,ab,kw OR (Weight-Lifting Exercise Program):ti,ab,kw OR (Weight-Lifting Strengthening Programs):ti,ab,kw (Word variations have been searched) 451

#10 #1 OR #2 144165

#11 #3 OR #4 2447

#12 #5 OR #6 OR #7 OR #8 OR #9 35366

#13 #10 and #11 and #12 460

WOS：

[((TS=(Sarcopenia OR sarcopenics OR strength OR lean mass OR muscle OR muscle mass)) AND TS=(Creatine OR creatine loading OR creatine supplementation OR creatine monohydrate)) AND TS=(resistance training OR training, resistance OR strength training OR training, strength OR weight-lifting strengthening program OR strengthening programs, weight-lifting OR strengthening program, weight-lifting OR weight lifting strengthening program OR weight-lifting strengthening programs OR weight-lifting exercise program OR exercise programs, weight-lifting OR exercise program, weight-lifting OR weight lifting exercise program OR weight-lifting exercise programs OR weight-bearing strengthening program OR strengthening programs, weight-bearing OR strengthening program, weight-bearing OR weight bearing strengthening program OR weight-bearing strengthening programs OR weight-bearing exercise program OR exercise programs, weight-bearing OR exercise program, weight-bearing OR weight bearing exercise program OR weight-bearing exercise programs ) | 1,405 检索结果](https://webofscience.clarivate.cn/wos/woscc/summary/5a1a78b3-f629-42c7-a4e5-754b421ceb9b-014c597496/relevance/1)

https://webofscience.clarivate.cn/wos/woscc/summary/5a1a78b3-f629-42c7-a4e5-754b421ceb9b-014c597496/relevance/1
